# A potential Chinese medicine monomer against influenza A virus and influenza B virus: isoquercitrin

**DOI:** 10.1186/s13020-023-00843-4

**Published:** 2023-11-02

**Authors:** Rongbo Luo, Chaoxiang Lv, Tiecheng Wang, Xiuwen Deng, Mingwei Sima, Jin Guo, Jing Qi, Weiyang Sun, Beilei Shen, Yuanguo Li, Donghui Yue, Yuwei Gao

**Affiliations:** 1 Changchun Veterinary Research Institute, Chinese Academy of Agricultural Sciences, Changchun, 130122 China; 2 The Research Center for Preclinical Medicine, Southwest Medical University, Luzhou, 646000 Sichuan China; 3 Jiangsu Co-Innovation Center for Prevention and Control of Important Animal Infectious Diseases and Zoonoses, Yangzhou University, Yangzhou, 225009 People’s Republic of China; 4 College of Integrated Chinese and Western Medicine, Changchun University of Chinese Medicine, Changchun, Jilin, 130117 China; 5 College of Life Sciences, Shandong Normal University, Jinan, 250014 China; 6 College of Life Sciences, Northeast Normal University, Changchun, 130021 China; 7 School of Medical Sciences, Changchun University of Chinese Medicine, Changchun, Jilin, 130117 China

**Keywords:** Influenza A virus, Influenza B virus, Chinese medicine monomer, Isoquercitrin, Cytokines, NF-κB signaling, Method of drug administration

## Abstract

**Background:**

Influenza viruses, especially Influenza A virus and Influenza B virus, are respiratory pathogens and can cause seasonal epidemics and pandemics. Severe influenza viruses infection induces strong host-defense response and excessive inflammatory response, resulting in acute lung damage, multiple organ failure and high mortality. Isoquercitrin is a Chinese medicine monomer, which was reported to have multiple biological activities, including antiviral activity against HSV, IAV, SARS-CoV-2 and so on. Aims of this study were to assess the in vitro anti-IAV and anti-IBV activity, evaluate the in vivo protective efficacy against lethal infection of the influenza virus and searched for the more optimal method of drug administration of isoquercitrin.

**Methods:**

In vitro infection model (MDCK and A549 cells) and mouse lethal infection model of Influenza A virus and Influenza B virus were used to evaluate the antiviral activity of isoquercitrin.

**Results:**

Isoquercitrin could significantly suppress the replication in vitro and in vivo and reduced the mortality of mouse lethal infection models. Compared with virus infection group, isoquercitrin mitigated lung and multiple organ damage. Moreover, isoquercitrin blocked hyperproduction of cytokines induced by virus infection via inactivating NF-κB signaling. Among these routes of isoquercitrin administration, intramuscular injection is a better drug delivery method.

**Conclusion:**

Isoquercitrin is a potential Chinese medicine monomer Against Influenza A Virus and Influenza B Virus infection.

**Supplementary Information:**

The online version contains supplementary material available at 10.1186/s13020-023-00843-4.

## Introduction

Influenza virus, belonging to the *Orthomyxoviridae* family, is a single-stranded, negative sense RNA virus and is one of the most significant pathogens threatening to human health. Influenza viruses are classified into four types (A, B, C, and D), of which influenza A virus (IAV) comprised the majority of global seasonal influenza case. Co-circulating with IAV, influenza B virus (IBV) is also responsible for about 25% of all influenza-related hospitalisations, especially cause high morbidity and mortality in children. [1, 2]. Continuing antigenic drift and shift of IAV and IBV results in new variants which facilitates the cross-species transmission and reduced efficacy of current vaccines and antiviral drugs. Thus, identifying new, more effective drugs against both IAV and IBV is of critical importance.

Isoquercitrin, also known as Quercetin-3-O-glucoside, is a kind of flavonol and widely exists in plants, like Houttuynia cordata, Hedyotis diffusa, et al. Currently, besides the direct extraction from plants, the chemical synthesis method was extensively used to prepare isoquercitrin. As a mono-glycoside derivative of quercetin which has diverse biological functions, isoquercitrin has been demonstrated to exhibit anti-tumor [3–5], anti-apoptotic [6], antioxidant [7] and anti-inflammatory [8] activities. Additionally, isoquercitrin also exerts antiviral activity against herpes simplex virus (HSV) [9, 10], influenza virus (IV) [11, 12], coronavirus (HCoV-229E, SARS-CoV-2) [13, 14], zika virus (ZIKV) [15, 16], mayaro virus (MAYV) [17], varicella-zoster virus (VZV) and human cytomegalovirus (HCMV) [18] in vitro. However, most of these studies have focused on in vitro studies and relatively few reports were performed on its in vivo treatment efficacy, especially against lethal infection of these viruses.

In this study, we found that isoquercitrin exhibited suppressive effect on influenza A (H1N1) virus and influenza B virus in vitro. Furthermore, isoquercitrin possesses a therapeutic effect in animal models through enhancing virus clearance, suppressing inflammation and attenuating lung pathological injury. Additionally, compared with other drug administrations, intramuscular injection is a better drug delivery method. These findings suggested that isoquercitrin might be a potential antiviral candidate against influenza A virus and influenza B virus.

## Materials and methods

### Cell culture, virus and compounds preparation

Human lung adenocarcinoma A549 cells and Madin-Darby canine kidney cells (MDCK) [19–21] were cultured in Dulbecco’s modified Eagle’s medium (DMEM) with 10% fetal bovine serum (FBS, Sigma), 1% penicillin and streptomycin. H1N1/UI182 strain was a mouse-adapted strain of the 2009 influenza A (H1N1) virus (A/Changchun/01/2009 (H1N1)). H1N1/PR8 virus, a laboratory adapted influenza A (H1N1) virus (A/Puerto Rico/8/34), was passaged in MDCK cells. Influenza B virus IBV/S9-E2 strain was rescued by Changchun Veterinary Research Institute according to the sequence of B/Yamagata/16/88 (GenBank Accession: CY018765-CY018772) and IBV/S9-MD stain was a mouse-adapted strain of IBV/S9-E2 strain. Isoquercitrin (CAS Number: 21637-25-2), purchased from Shanghai yuanye Bio-Technology Co., Ltd, was dissolved in dimethyl sulfoxide (DMSO) and diluted in DMEM with 2% FBS or Phosphate Buffered Saline (PBS, pH 7.4) before use.

### Cytotoxicity assay, in vitro antiviral assay and time-of-addition assay

MDCK and A549 cells were seeded in 96-well plate (10^4^ cells/well) and incubated at 37 °C with 5% CO_2_. When the confluence reached 70–80%, different concentrations of the drug (8, 15, 30, 60, 125, 250 μg/mL) were added. After 48 h, cell viability was measured by CCK-8 assay using Cell Counting Kit-8 (Beyotime, C0039) following manufactory’s instruction. For in vitro antiviral assay, cells were washed with PBS and infected with influenza A virus (H1N1/PR8 and H1N1/UI182) and influenza B virus (IBV/S9-E2 and IBV/S9-MD) (MOI = 0.1) when cell confluence reached 70–80%. After 1 h post infection, isoquercitrin were added. After 48 h, cell viability was measured by CCK-8 assay. As for time-of-addition assay, cells were treated with isoquercitrin for 1 h before virus infection or inoculated with isoquercitrin-viruses mixture, after 1 h virus adsorption. At 48 h post-infection, cell viability was measured by CCK-8 assay.

### Immunofluorescence staining

The cells were seeded in 12-well plates and infected with H1N1/UI182 (MOI = 0.5) when confluence reached 70%. At 48 h post-infection, cells were washed three times with cold PBS and fixed with 4% paraformaldehyde (PFA) for 20 min, then the plate was infiltrated with 0.2% Triton-X100 and blocked with 2% BSA for 1 h. The primary antibody against influenza virus Nucleocapsid protein (Abcam, ab104870, 1:200) was added and incubated with cells overnight at 4 ℃. After the primary antibodies, cells were washed three times using PBS and incubated with the secondary antibodies (Goat Anti-Rabbit IgG H&L (Alexa Fluor^®^ 488), Abcam, ab150077, 1:500) for 2 h without light. After nuclei staining with Hoechst 33,258 (Thermo Fisher Scientific, H3569, 1 µg/mL) for 10 min, fluorescence was observed under a fluorescence microscope (Carl Zeiss, Germany).

### In vivo experiments in mice

Female BALB/c mice (18–20 g, 6 weeks old) were purchased from Beijing Vital River Laboratory Animal Technology Co., Ltd (Beijing, China). After 3 days of adaptive feeding, mice were randomly divided into uninfected group (Control), virus-infected groups (H1N1/PR8 and IBV/S9-MD), isoquercitrin-treated group, oseltamivir phosphate (a NA inhibitor)-treated group. Mice in each group, except uninfected group, were challenged with 50 µL virus dilution (10 × LD_50_) using nasal drop method, respectively. Control group and virus-infected groups were treated with 0.9% saline by intraperitoneal injection (i.p.). The isoquercitrin treatment (10 mg/kg/day, i.p) [22] and oseltamivir phosphate treatment (25 mg/kg/day, p.o.) [23] started after 12 h post-infection and until the fifth day. Mouse body weight and survival status were monitored each day for 14 days.

### Pathological analysis and immunohistochemical assay

At 3 day post-infection (3 dpi) and 5 day post-infection (5 dpi), lung, heart, liver, spleen and kidney of mice in each group were collected, part of tissue was used for analysis of gene and protein expression, part of tissue was fixed in the 4% paraformaldehyde (PFA), preparing for tissue sections. For pathological analysis, tissue sections were stained with hematoxylin and eosin (H&E), dried and observed under a light microscope (Olympus, Japan). Immunohistochemical assay was performed as described previously [47].

### RNA isolation and quantitative RT-PCR

Total RNA from mouse lung tissues of mice was extracted using the HiPure Universal RNA Kit (Magen, R4130-03). About 1 μg RNA was used to synthesize cDNA using PrimeScript^™^ RT reagent Kit (Takara, RR047A). The expression level of target mRNA was measured by qRT-PCR assay using TB Green Premix Ex Taq II (Takara, RR820A) based on TB Green chimeric fluorescence method. The primer pairs were listed in Table 1, *β-actin* was used as a loading control.

**Table 1:** The sequence of primer for qRT-PCR

Gene name	Primer sequence (5′ to 3′)
β-actin (mouse)	F: 5′—TGACGTTGACATCCGTAAAGACC-3′
	R: 5′—AAGGGTGTAAAACGCAGCTCA-3′
IFN-α (mouse)	F: 5′—GCACCCTGCCTCAGACTCAC-3′ R: 5′—TGCCTGGTCATCTCATGGAAG-3′
IFN-β (mouse)	F: 5′—TGCATCTTCTCCGTCATCTC-3′ R: 5′—TAGCAGCCGACACCAGCCTG-3′
TNF-α (mouse)	F: 5′—AGCCCTGGTATGAACCCATC-3′ R: ′—GGAATCGGCAAAGTCAAGGT-3′
IL-1β (mouse)	F:5′—TCATCGTGGCAGTGGAAAAG-3′ R: 5′—GGGAAGCAAGGGTCTCAGGT-3′
IL-6 (mouse)	F: 5′—AGTTGCCTTCTTGGGACTGATG-3′ R: 5′—GGGAGTGGTATCCTCTGTGAAGTCT-3′
IL-10 (mouse)	F: 5′—TGACCCAGACATCAAGGAACAT-3′ R: 5′—GTCAAACTCACTCATGGCTTTGTA-3′
ISG54 (mouse)	F: 5′—CACCTCTGGACTGGCAATAGC-3′ R: 5′—GTCAGGATTCAGCCGAATGG-3′
ISG56 (mouse)	F: 5′—TCATCAGGTCAAGGATAGTC-3′ R: 5′—CCACACTGTATTTGGTGTCTAGG-3’

### EID_50_ detection

9 day-old embryonated chicken eggs were used to detect EID_50_ (50% egg-infective dose) of virus. An equivalent number of mouse lung tissues from each group were crushed in DMEM supplemented with 400 units/mL penicillin and streptomycin. Followed by centrifugation, the supernatant was serially diluted tenfold from 10^–1^ to 10^–8^ and were inoculated into eggs for 48 h at 37 ℃ (for IBV, eggs should incubate for 72 h at 33 ℃). Then 50 μL allantoic fluid in each egg was collected and mixed with 50 μL 1% chicken RBC. After standing for 15 min, the highest dilutions that completely agglutinated chicken RBC were recorded. Viral titer was calculated by the Reed-Muench method, and the result was expressed as log_10_ EID_50_.

### Western blot

The mouse lung tissues from each group were lysed with RIPA Lysis Buffer (Beyotime, P0013B) to collect total proteins. After protein quantification and electrophoresis, the proteins were transferred on a PVDF membrane. The protein expression was detected by relative antibody and β-actin (Abcam, ab6276, 1:5000) was used as an internal reference protein for quantitative analysis.

### Statistical analysis

Statistical comparisons were performed using ANOVA analysis. Quantitative data sets were expressed as the means ± standard error (SE), and the statistical significance was evaluated by Graphpad Prism 8.0 software. Compared with the Virus-infected group, P < 0.05 was regarded to have significant differences.*P < 0.05, **P < 0.01, and ***P < 0.001.

## Results

### Isoquercitrin inhibits influenza virus in vitro

**Fig. 1:**
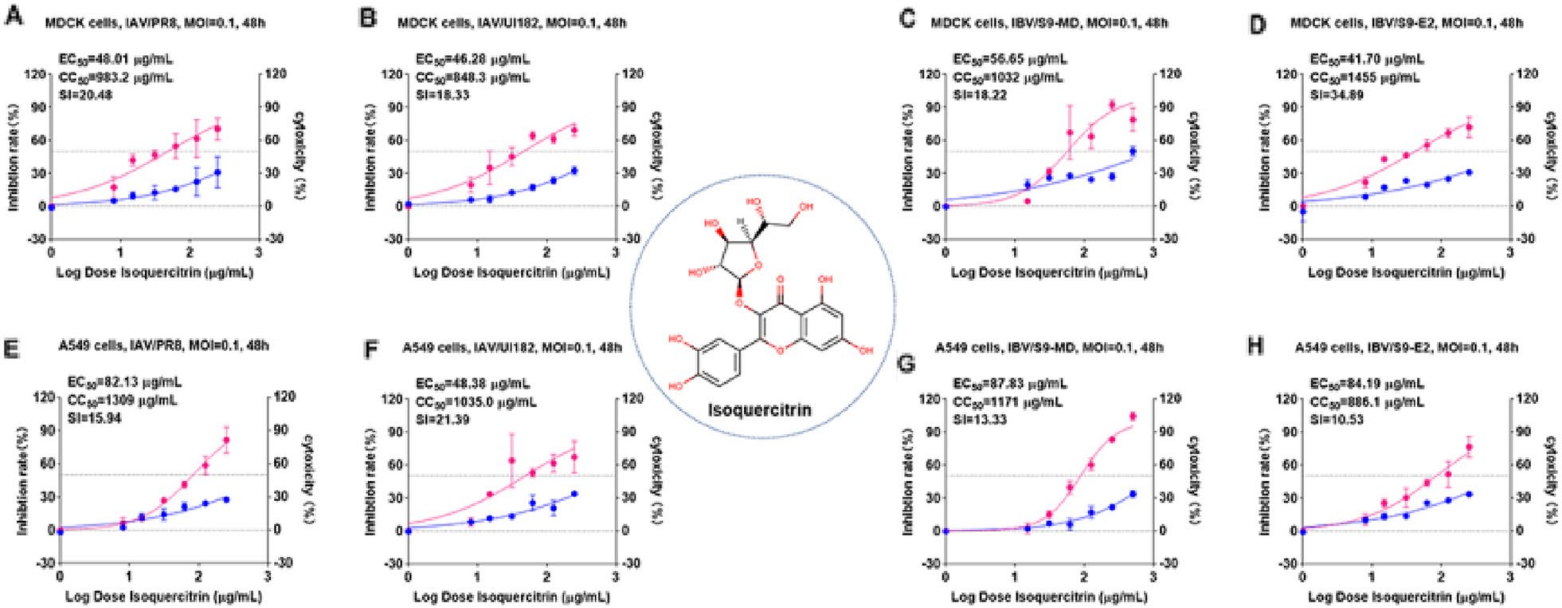
Isoquercitrin inhibits influenza virus in vitro. The inhibitory efects of isoquercitrin on the influenza A **A**, **E**-PR8, **B**, **F**-UI182) virus and influenza B **C**, **G** S9-MD, **D**, **H**-S9-E2) virus in MDCK cells **A**–**D** and A549 cells **E**–**H**. As shown in the figure, pink curve represents the dose-dependent relationship between drug inhibition rate against virus and dosage, blue curve represents the dose-dependent relationship between drug’s cytotoxicity and dosage. Three independent experiments were repeated and the figure showed the results of one representative experiment out of three performed. The EC_50_/IC_50_ values in the figure were obtained through curve fitting using Graphpad Prism 8.0 software and only represented the data from this experiment. The SI indicated the selection index (SI = IC_50_/EC_50_) of isoquercitrin

**Fig. 2:**
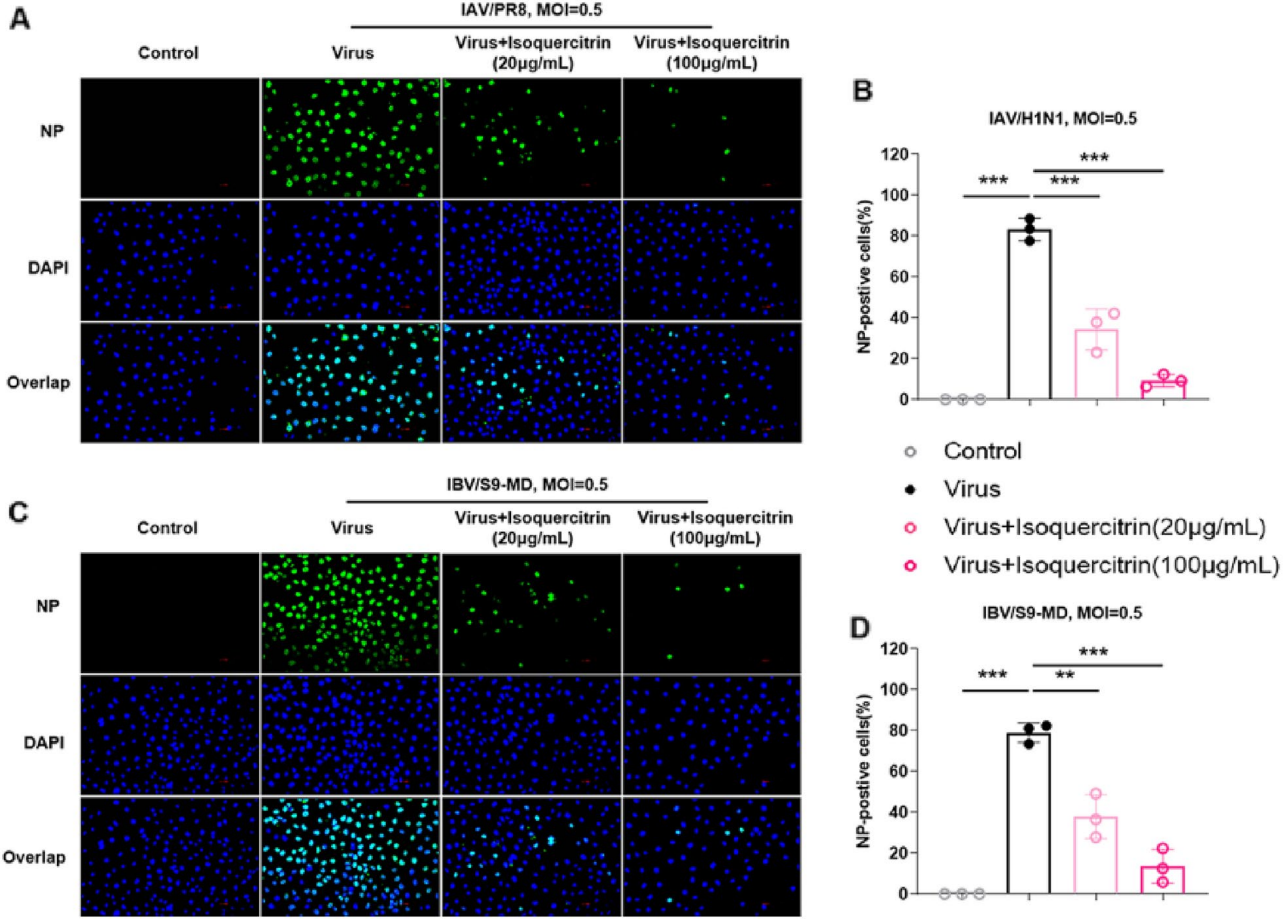
Isoquercitrin inhibits influenza virus in vitro. The effect of isoquercitrin on the expression of influenza A (H1N1/PR8) virus **A** and influenza B (S9-MD) virus **C** proteins NP in infected MDCK cells at low dose (20 μg/mL) and high dose (100 μg/mL). **B**, **D** The percentage of NP-positive cells in **A** and **B** was calculated

To determinate whether isoquercitrin can inhibit different subtypes of influenza virus, the antiviral assays were performed in vitro against IAV and IBV. We first assessed antiviral activity of isoquercitrin in MDCK and A549 cells. Cells, infected with or without virus, were treated with isoquercitrin at different concentrations for 48 h, and cell proliferation was assessed using CCK-8 assay. The results showed that isoquercitrin inhibited IAV and IBV infection without inducing significant cytotoxicity (CC_50_ > 800 μg/mL), with EC_50_ of 46.77 ± 1.31 μg/mL for H1N1/PR8 (Fig. 1A), 46.52 ± 4.70 μg/mL for H1N1/UI182 (Fig. 1B), 48.90 ± 7.49 μg/mL for IBV/S9-MD (Fig. 1C) and 42.63 ± 3.64 μg/mL for IBV/S9-E2 (Fig. 1D) in MDCK cells. Likewise, isoquercitrin blocked IAV and IBV infection in A549 cells, with selectivity index (SI = CC_50_/EC_50_) higher than 10 (Fig. 1E–H). To investigate which stage of influenza virus infection does isoquercitrin exert the antiviral effect, time-of-addition assay was performed. The inhibitory effect of different drug administration protocols showed that isoquercitrin played an antiviral role main in late stage of influenza virus infection, as treatment after infection exerted the maximum antiviral effect against both IAV and IBV (Additional file 1: Fig. S1). Additionally, the results of immunofluorescence experiments indicated that isoquercitrin suppressed Nucleoprotein (NP) expression of IAV (Fig. 2A, B) and IBV (Fig. 2C, D) in MDCK cells. Taken together, these data showed that isoquercitrin could potently inhibit both IAV and IBV infection in different cell lines.

### Isoquercitrin protects mice from a lethal infection of the influenza virus

**Fig. 3:**
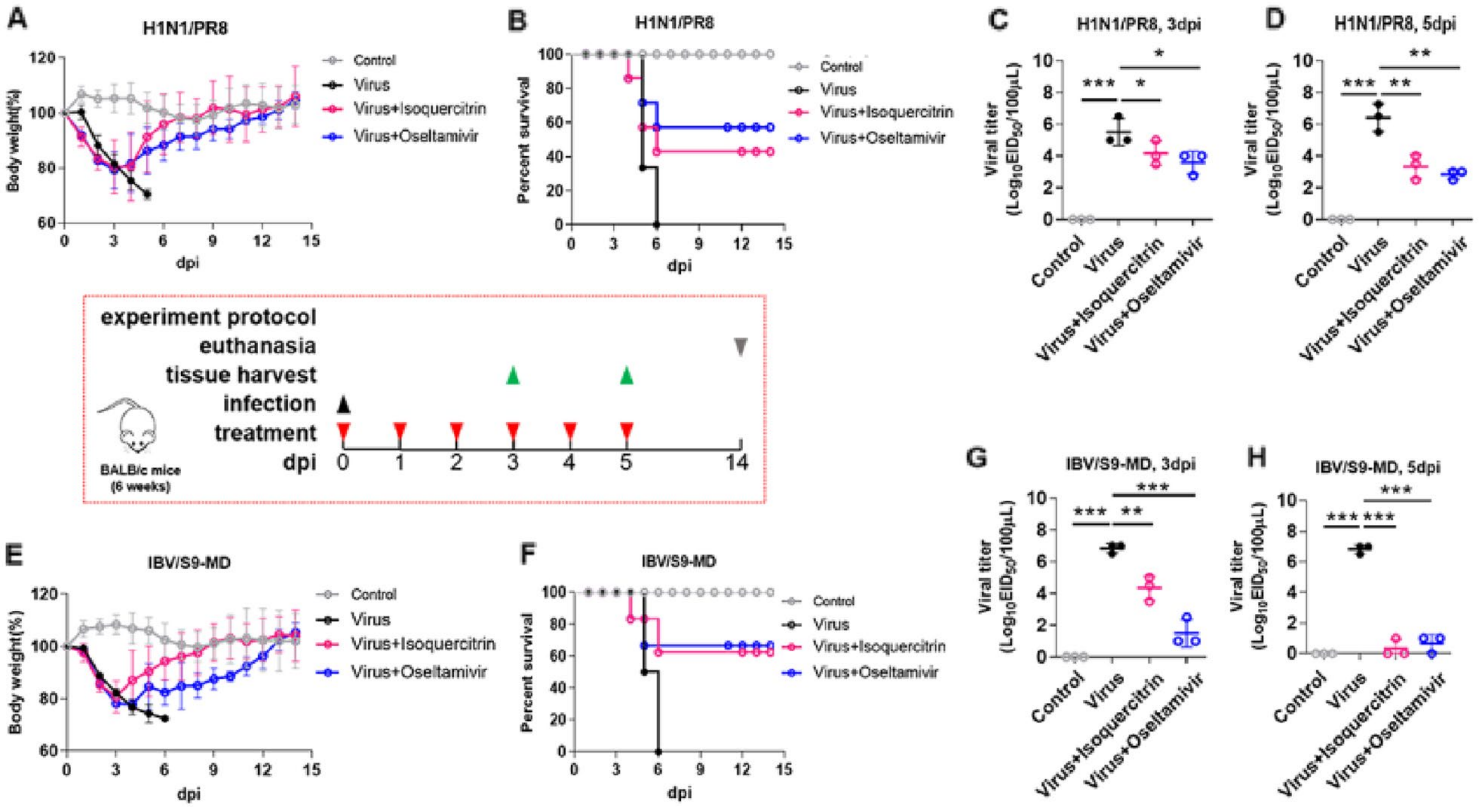
Isoquercitrin protects mice from a lethal infection of the influenza virus. **A** Body weight changes of H1N1/PR8-infected mice and drug-treated mice. **B** Survival status of H1N1/PR8-infected mice and drug-treated mice. **C**–**D** The effects of isoquercitrin and oseltamivir phosphate on viral titer in mouse lungs at 3 dpi and 5 dpi. **E** Body weight changes of IBV/S9-MD-infected mice and drug-treated mice. **F** Survival status of IBV/S9-MD-infected mice and drug-treated mice. **G**–**H** The effects of isoquercitrin and oseltamivir phosphate on viral titer in mouse lungs at 3 dpi and 5 dpi

To assess the protection efficacy of isoquercitrin in a mouse lethal infection model, BALB/c mice were challenged with a lethal dose of influenza A virus (H1N1/PR8) and influenza B virus (IBV/S9-MD). After corresponding treatment, four mice of each group were euthanized and tissues (lung, heart, liver, spleen, kidney) were collected for further analysis. The remained mice are used to monitor the body weight change and survival status. The results showed that virus infection induced obvious reduction of mouse body weight from day 2 and all mice in H1N1/PR8-infected group and IBV/S9-MD-infected group dead by day 6 after infection. In contrast, isoquercitrin and oseltamivir treatment attenuated weight loss and increased the survival rate of infected mice. Surprisingly, isoquercitrin significantly promoted mouse body weight recovery, which was better than oseltamivir, but its protective effect was inferior to Oseltamivir (Fig. 3A, B, E, F). Meanwhile, the effect of isoquercitrin on viral titer in mouse lungs was determined at 3 dpi and 5 dpi. The results showed that viral titer of IAV (Fig. 3C, D) and IBV (Fig. 3G, H) was significantly reduced by isoquercitrin. These results reveal that isoquercitrin has a potently inhibitory effect against H1N1/PR8 and IBV/S9-MD, and protects mice from a lethal infection of influenza virus.

### Effect of isoquercitrin on pathological damage caused by influenza virus infection

**Fig. 4:**
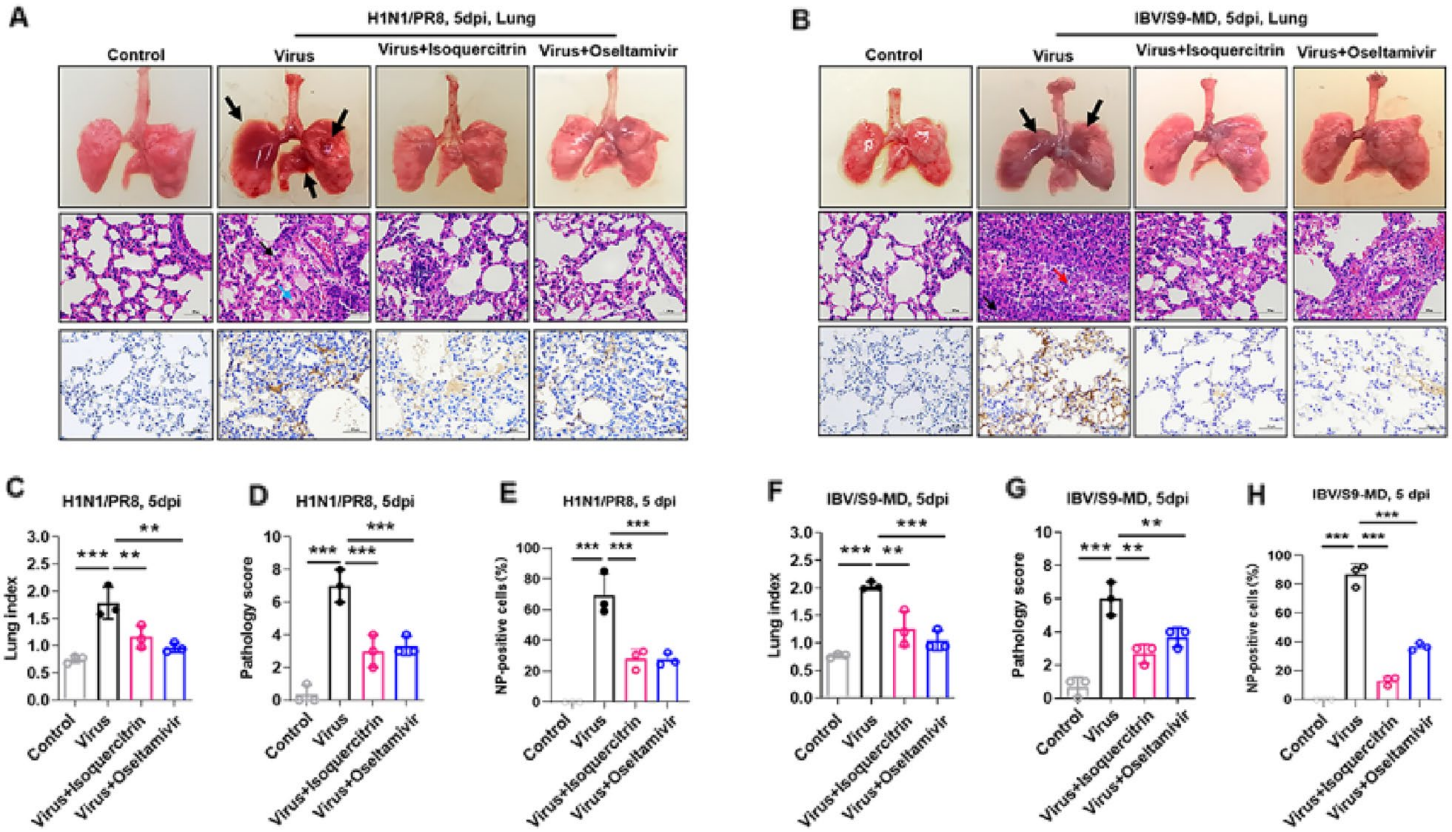
Effect of isoquercitrin on pathological damage caused by influenza virus infection. **A** Lung morphology, H and E staining and Immunohistochemistry staining of virus protein NP in H1N1/PR8-infected mice and drug-treated mice at 5 days post-infection (dpi). **B** Lung morphology, H and E staining and Immunohistochemistry staining of virus protein NP in IBV/S9-MD-infected mice and drug-treated mice at 5 dpi. **C**–**E** Lung index, lung pathology score and percentage of NP-positive cell in lung tissues in **A**. **F**–**H** Lung index, lung pathology score and percentage of NP-positive cell in lung tissues in **B**

Influenza virus infection can cause variable degree of lung injury in host which is an important standard of therapeutic efficacy of antiviral drugs [24]. Thus, we evaluated the effect of isoquercitrin on influenza virus-induced pathological damage in mouse infection models. Morphologically, H1N1/PR8 and IBV/S9-MD infection caused wade-range of visible hemorrhage and lesions on surface of the lungs and these symptoms were alleviated after treatment with isoquercitrin and oseltamivir. (Fig. 4A, B). H and E staining of the lung tissues showed that after infection with influenza virus, thickened alveolar walls were observed, accompanied by necrotic cell debris in partial bronchiolar lumen (red arrow), infiltration of a large number of lymphocytes and neutrophils (black arrow), and effusion of eosinophilic material within alveolar spaces (blue arrow), and these damages were reversed by isoquercitrin and oseltamivir. Furthermore, analysis of lung index (Fig. 4C, F) and lung pathology score (Fig. 4D, G) of mice in each group visually revealed the potent protective effects of isoquercitrin and oseltamivir against H1N1/PR8 and IBV/S9-MD infection. The results of viral NP immunohistochemistry (IHC) (Fig. 4E, H) showed that isoquercitrin significantly inhibited influenza virus nucleoprotein (NP) expression in lung tissues. Similarly, isoquercitrin also attenuated pathological damage (Additional file 1: Fig. S2A) and decreased the percentages of NP-positive cells (Additional file 1: Fig. S2B, C) in mouse heart, liver, spleen, kidney. Together, these results suggest that isoquercitrin suppresses the viral protein expression and mitigates influenza virus-caused pathological damage in major organs of mice.

### Isoquercitrin inhibits cytokines induced by influenza virus infection by inactivating NF-κB signal

**Fig. 5:**
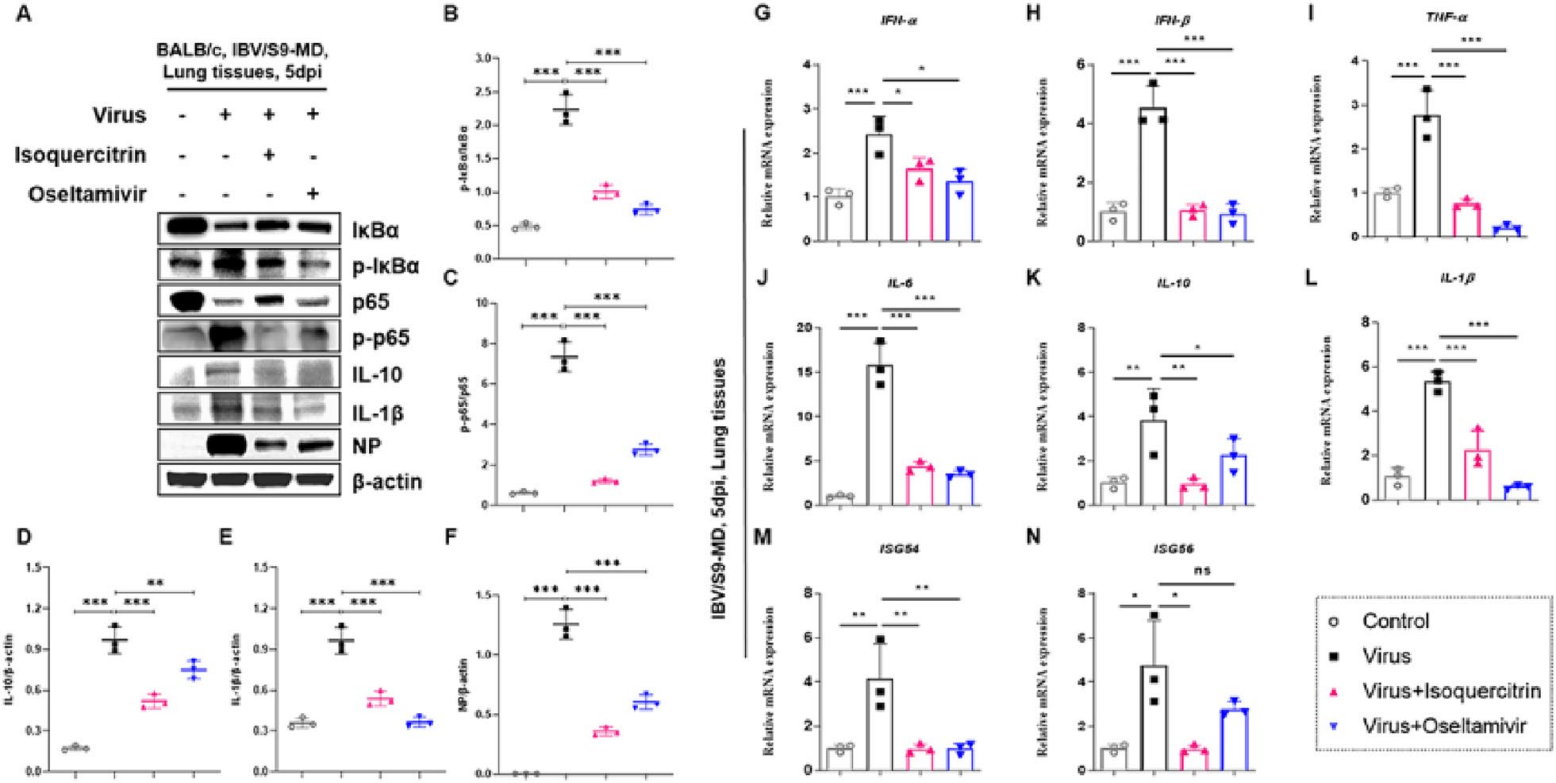
Isoquercitrin inhibits cytokines expression induced by influenza B virus infection by inactivating NF-κB signal. At 5 days post infection, mouse lung tissues were harvested for Western blot experiments and qPCR analysis. **A** Western blot images of IκBα, phosphorylated IκBα (p-IκBα), NF-κB (p65), p-p65, IL-10, IL-1β, viral Nucleoprotein (NP) and β-actin was shown. **B** p-IκBα/IκBα ratio was quantified. **C** p-p65/p65 ratio was quantified. **D** Quantification of Western blot bands of IL-10. **E** Quantification of Western blot bands of IL-1β. **F** Quantification of Western blot bands of viral NP. Relative mRNA expression level of type I IFN, *IFN-α* **G** and *IFN-β* **H**, pro-inflammatory cytokines, *TNF-α* **I**, *IL-6* **J** and *IL-1β* **L**, anti-inflammatory cytokine, *IL-10* **K** and IFN induced gene, ISG54 **M** and *ISG56* **N** in mouse lung tissues of each group were detected

The activation of innate immune cells and the production of cytokines are important markers of influenza virus infection. Continuous generation of cytokines exacerbates the influenza virus-induced “cytokine storm”, which is main cause of lung damage of host [25]. To evaluate the effect of isoquercitrin on the cytokines production, Western blot and quantitative real-time PCR assay (qPCR) were performed. The results of Western blot (Fig. 5A, F) revealed that IBV/S9-MD infection increased protein expression of inflammatory factors, IL-10 (Fig. 5D) and LI-1β (Fig. 5E) in mouse lung tissues. Similarly, qPCR results showed that a lethal dose of IBV/S9-MD infection could significantly upregulated mRNA expression of cytokines, *IFN-α* (Fig. 5G), *IFN-β* (Fig. 5H), *TNF-α* (Fig. 5I), *IL-6* (Fig. 5J), *IL-10* (Fig. 5K), and *IL-1β* (Fig. 5L) and interferon-stimulated gene, *ISG54* (Fig. 5M) and *ISG56* (Fig. 5N), but this induction was potently suppressed by isoquercitrin and oseltamivir. Meanwhile, isoquercitrin also had a significantly inhibitory effect on cytokines production caused by H1N1/PR8 infection (Additional file 1: Figure S3), with a similar or even better inhibition than oseltamivir. NF-κB pathway plays as a major regulator of cytokine and chemokine expression after influenza virus infection and also influences influenza virus replication [26]. Thus, the protein expression of phosphorylated NF-κB (p65) and IκBα were detected. Western Blot results (Fig. 5A) indicated that isoquercitrin could reduced the phosphorylation of IκBα (Fig. 5B) and p65 (Fig. 5C) which were up-regulated by influenza B virus, resulting the inactivity of NF-κB signaling. These findings demonstrate that isoquercitrin is able to suppress the inflammatory response induced by influenza virus infection via inactivating NF-κB signaling.

### Intramuscular injection is a better drug delivery method of isoquercitrin

**Fig. 6:**
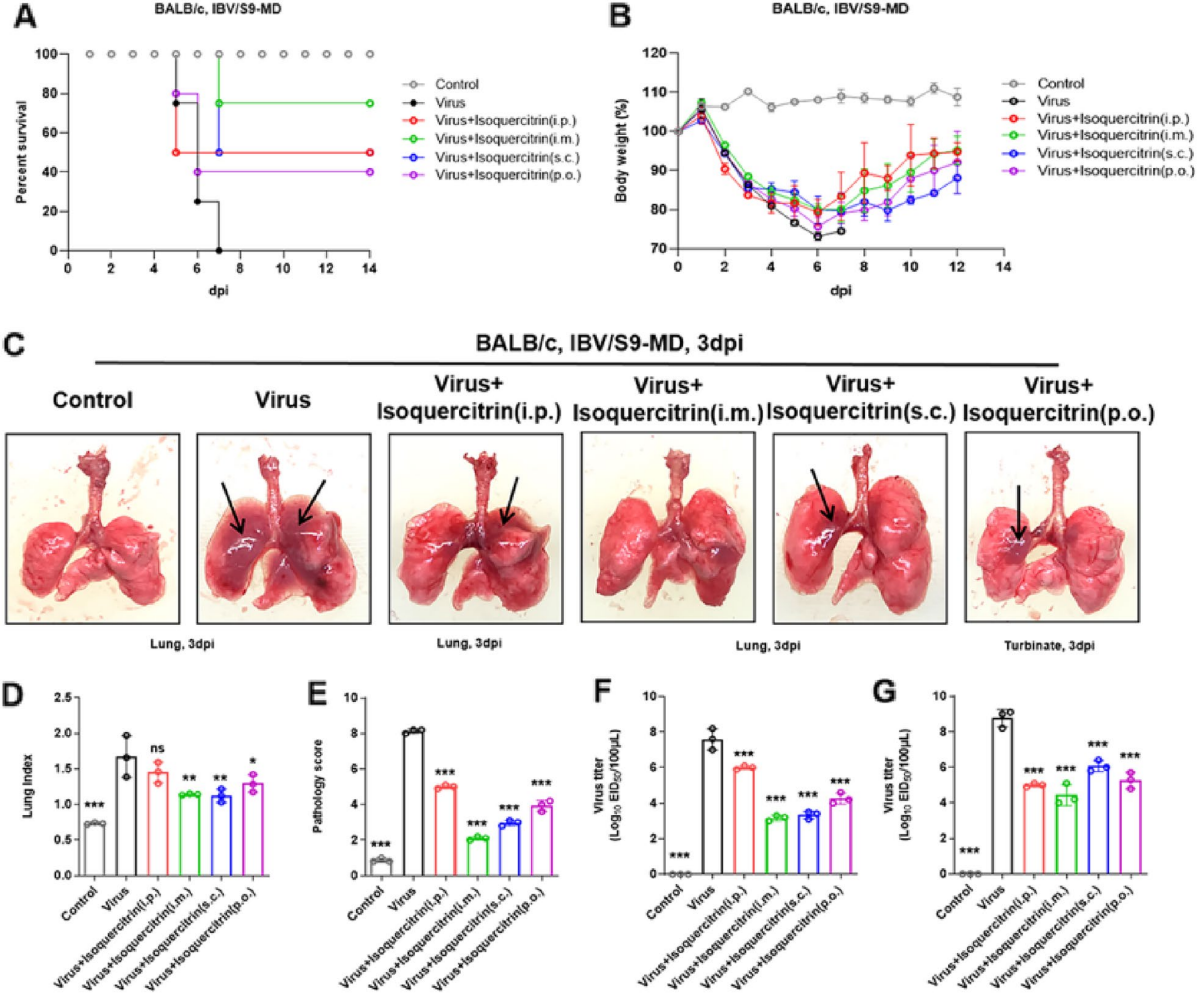
The comparison of antiviral efficacy of different drug delivery method of isoquercitrin. **A** Survival status of mice in each group. **B** Body weight changes of mice in each group. **C** Lung morphology of mice in each group at 3 dpi. **D** Lung index of mice in each group. **E** Lung pathology score of mice in each group. **F** Virus titer in lung tissues of mice in each group. **G** Virus titer in turbinates of mice in each group

Mode of drug administration affects absorption, distribution, metabolism and ultimately drug efficacy. To compare the antiviral effect of different drug delivery method of isoquercitrin, lethal mouse model of IBV infection was treated with isoquercitrin using different modes of drug administration, including intraperitoneal injection (i.p.), intramuscular injection (i.m.), oral gavage (p.o.), and subcutaneous injection (s.c.). The results displayed in Fig. 6 shown that isoquercitrin treatment sped up weight restoration of infected mice (Fig. 6B), and improved the survival rate (Fig. 6A). Noticeably, treated by the i.m. could achieve better therapeutic outcomes compared with other drug delivery methods, providing better improvement of mouse lung damage (Fig. 6C–E) and more significant reduction in virus titers (Fig. 6F–G). These results indicate that intramuscular injection is a better method of administration for isoquercitrin.

## Discussion

IAV infection is main causes of seasonal influenza epidemics and pandemics which can induce human respiratory disease and acute lung injury (ALI). Meanwhile, IBV often co-circulates with IAV and even becomes major epidemic strains worldwide [27]. Existing antiviral strategies to control emerging influenza strains mainly focus on vaccines and antiviral drugs development. However, frequent antigenic drift and antigenic switch of IAV generates novel strains which can escape from available vaccines and reduce efficiency of antiviral drugs. For example, 100% of seasonal H3N2 and 99.8% of 2009 pandemic influenza samples was found resistant to adamantanes, M2 inhibitor [28]. Similarly, due to mutation at particular amino acid, NA inhibitors-resistant human isolates gradually emerged, like H1N1 (NA-I222V/M/L/R/T, H274Y and S334N) [29], H3N2 (NA-E119V, E11V) [30], H7N9 (NA-R292K) [31] and IBV (NA-G104R) [32]. Given that IBV also has the antigenic drift and can develop drug-resistance, identifying and developing new antiviral drugs is imperative. In our study, although our drug has weaker in vitro antiviral effects than oseltamivir, whose EC_50_ values for influenza A virus were in the low nanomolar (~ 2–13 nM) concentration range [33], but in vivo, 10 mg/kg/day of isoquercitrin had equivalent efficacy to 25 mg/kg/day of oseltamivir phosphate, suggesting this drug is a better in vivo therapeutic drug. Given the continuous emergence of resistance of oseltamivir, perhaps isoquercitrin can become a new treatment option for drug-resistant strains of influenza A virus and influenza B virus.

Isoquercitrin is a flavonoid that can be extracted from natural plants and exhibits antiviral activity against multiple viruses. Isoquercitrin was reported to be the main component of some Traditional Chinese medicine (TCM) that suppressed HSV by inhibiting ROS production and NF-κB activation [9, 10], suppressed IAV (H1N1) replication by inhibiting ROS production [11] and exerted antiviral activity against SARS-CoV-2 [34] and HCoV-229E [13] by blocked Spike protein and Mpro. Isoquercitrin also blocked ZIKV infection through reduced ZIKV NS2B-NS3 protease and NS5 RNA dependent RNA polymerase (RdRp) activity in cells and *Ifnar1*^−/−^ mice [16, 35]. Taken its inhibitory effect on many RNA viruses and even DNA viruses and results of our researches, isoquercitrin could become a broad-spectrum antiviral candidate.

Cytokine is a kind of peptide or glycoprotein secreted by the cells and perform diverse biological functions, such as immune modulation, involvement in inflammatory responses, etc. Cytokine is a crucial component of antiviral innate immune, but severely viral infections are always accompanied by cytokine dysregulation and amplification, even becomes the main factor for viral pneumonia during the later phase after virus infection [36]. Considering that, reducing cytokine production is a potential treatment strategy. Thus, multiple immunomodulators, such as Janus kinase (JAK) inhibitors [37] and anti‐IL‐6 antibody [38], are developed to control virus-induced “cytokine storm” and mitigate disease progression. In addition to chemical molecules and antibodies, traditional Chinese medicine gradually become an alternative source for anti-influenza virus drugs [39]. Fructuscorni and Radix salviae miltiorrhizae possess resistance to influenza because they can enhance specific and non-specific immunity [40]. As a natural drug, isoquercitrin was reported to reduce the virus-induced ROS production and NF-κB activation [10]. Taken together, isoquercitrin not only has antiviral activity against IAV and IBV, but also represses the expression of pro-inflammatory cytokines by inactiviating NF-κB signal.

The pathogenicity of an influenza virus depends on many factors, such as cell tropism, replicate efficiency, and host immunity. Both IAV and IBV prefer to replicate in respiratory system and innate immune cells are recruited to lung and produce massive cytokines and chemokines after influenza virus infection, inducing pneumonia, even multi-organ failure [41]. Unexpectedly, adolescents and children are more susceptible to IBV and sometimes IBV-associated diseases develop earlier and more severe than IAV [42]. Characterized by inflammatory cell infiltration, alveolar epithelial cells sloughing, alveolar wall hyperemia and thickness, alveolar hemorrhage and damage, interstitial-alveolar edema, etc. the degree of which always becomes a standard for assess the pathogenicity of viruses and the efficacy of therapeutic drugs [43]. Fortunately, in this study, isoquercitrin was found as a treatment drug for viral pneumonia.

The dose and route of administration are main factors affecting the drug efficacy [44]. In other researches, isoquercitrin was demonstrated to exert therapeutic effect at lower doses, so we only explore its dose-dependent relationship. In a murine model, the common route for drug administration include tail-vein injection, intraperitoneal injection, intramuscular injection, subcutaneous injection, oral gavage and intranasal administration. Compared to tail-vein injection, which can increase the risk of infection and thromboembolic events [45], intraperitoneal injection, intramuscular injection and subcutaneous injection are more safe and long-acting. Oral administration is the predominant method of drug administration of natural products [46], but its bioavailability should be considered. In our study, isoquercitrin treatment via different administration could exert antiviral effect, suggesting that this drug may directly exert the pharmacodynamic effect in target organ, without requirement of metabolic conversion. Additionally, considering the influence of different drug administration on the antiviral effect of isoquercitrin, more experiments should be considered be implemented to identify the optimal dosing regimen.

## Conclusion

Above findings suggest that isoquercitrin has potent antiviral activity against influenza A (H1N1) virus and influenza B virus in vitro and in vivo. Lethal infection of influenza virus induced excessive production of cytokines and severe lung damage, but these outcomes were suppressed by isoquercitrin, indicating that this drug could benefit severe patients with influenza virus infection. Additionally, through comparison between different drug delivery methods, the i.m. was proved as a better method for administration of isoquercitrin.

### Supplementary Information

**Additional file 1: Fig. S1.** The effect of timing of drug administration and dose on anti-IAV **A**–**B** and anti-IBV **C**–**D** activity of isoquercitrin in MDCK cells and A549 cells. Colors annotate three drug administration protocols, pre-treatment (gray), co-treatment (black) and post-treatment (pink). **Fig. S2.** Effect of isoquercitrin on pathological damage caused by influenza virus infection. **A** HE staining of heart, liver, spleen and kidney tissues in influenza virus-infected mice and drug-treated mice at 5 dpi. **B** Immunohistochemistry staining of heart, liver, spleen and kidney tissues in influenza virus-infected mice and drug-treated mice at 5 dpi. **C** The percentage of NP positive cells in main tissues (heart, liver, spleen, kidney) in **B**. **Fig. S3.** Isoquercitrin inhibits cytokines induced by influenza A virus infection. Relative mRNA expression level of type I IFN (*IFN-α* and *IFN-β*), pro-inflammatory cytokines (*TNF-α*, *IL-6* and *IL-1β*), anti-inflammatory cytokine (*IL-10*) and IFN induced gene (ISG54 and *ISG56*) in mouse lung tissues of each group at 5 dpi.

## Data Availability

The data produced from this study are available from the first author and the corresponding author on reasonable request.
