# Supplementary material for: A potential Chinese medicine monomer against influenza A virus and influenza B virus: isoquercitrin
Source: Chin Med. 2023 Nov 2;18:144. doi: 10.1186/s13020-023-00843-4 (PMC10621105; doi:10.1186/s13020-023-00843-4)

**Fig. S1** The effect of timing of drug administration and dose on anti-IAV (**A-B**) and anti-IBV (**C-D**) activity of isoquercitrin in MDCK cells and A549 cells. Colors annotate three drug administration protocols, pre-treatment (gray), co-treatment (black) and post-treatment (pink).

**Fig. S2** Effect of isoquercitrin on pathological damage caused by influenza virus infection. (**A**) HE staining of heart, liver, spleen and kidney tissues in influenza virus-infected mice and drug-treated mice at 5 dpi. (**B**) Immunohistochemistry staining of heart, liver, spleen and kidney tissues in influenza virus-infected mice and drug-treated mice at 5 dpi. (**C**) The percentage of NP positive cells in main tissues (heart, liver, spleen, kidney) in (**B**).

**Fig. S3** Isoquercitrin inhibits cytokines induced by influenza A virus infection. Relative mRNA expression level of type I IFN (*IFN-α* and *IFN-β*), pro-inflammatory cytokines (*TNF-α*, *IL-6* and *IL-1β*), anti-inflammatory cytokine (*IL-10*) and IFN induced gene (ISG54 and *ISG56*) in mouse lung tissues of each group at 5 dpi.


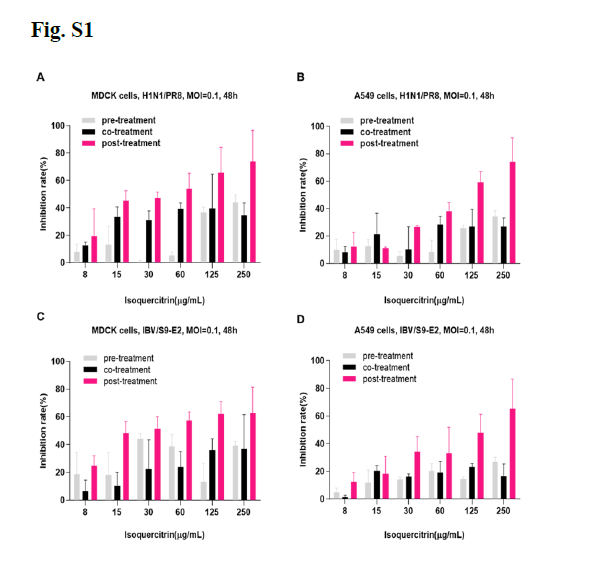


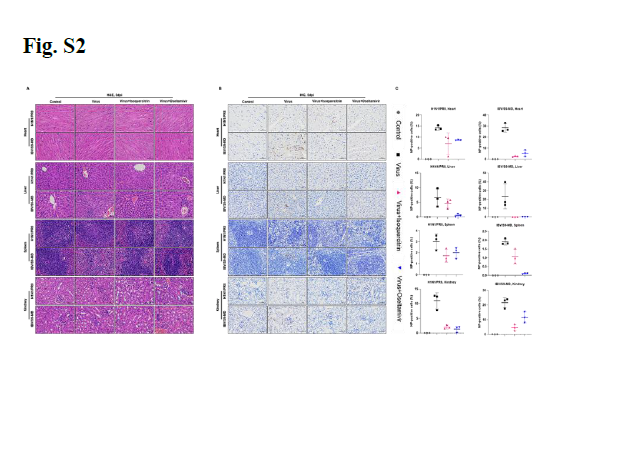


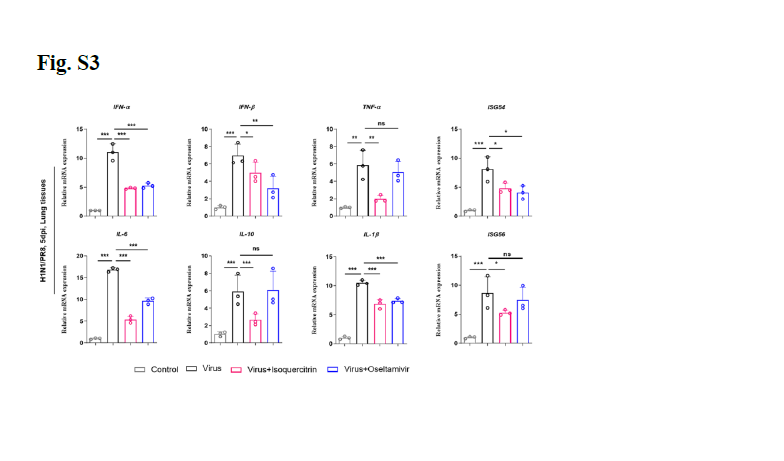

Supplement: Supplementary file 1 — Additional file 1: Fig. S1. The effect of timing of drug administration and dose on anti-IAV A–B and anti-IBV C–D activity of isoquercitrin in MDCK cells and A549 cells. Colors annotate three drug administration protocols, pre-treatment (gray), co-treatment (black) and post-treatment (pink). Fig. S2. Effect of isoquercitrin on pathological damage caused by influenza virus infection. A HE staining of heart, liver, spleen and kidney tissues in influenza virus-infected mice and drug-treated mice at 5 dpi. B Immunohistochemistry staining of heart, liver, spleen and kidney tissues in influenza virus-infected mice and drug-treated mice at 5 dpi. C The percentage of NP positive cells in main tissues (heart, liver, spleen, kidney) in B. Fig. S3. Isoquercitrin inhibits cytokines induced by influenza A virus infection. Relative mRNA expression level of type I IFN (IFN-α and IFN-β), pro-inflammatory cytokines (TNF-α, IL-6 and IL-1β), anti-inflammatory cytokine (IL-10) and IFN induced gene (ISG54 and ISG56) in mouse lung tissues of each group at 5 dpi. [file 13020_2023_843_MOESM1_ESM.docx]
